# Improving patient flow during infectious disease outbreaks using machine learning for real-time prediction of patient readiness for discharge

**DOI:** 10.1371/journal.pone.0260476

**Published:** 2021-11-23

**Authors:** Jennifer A. Bishop, Hamza A. Javed, Rasheed el-Bouri, Tingting Zhu, Thomas Taylor, Tim Peto, Peter Watkinson, David W. Eyre, David A. Clifton

**Affiliations:** 1 Department of Engineering Science, University of Oxford, Oxford, United Kingdom; 2 Oxford University Hospitals NHS Foundation Trust, Oxford, United Kingdom; 3 Big Data Institute, Nuffield Department of Population Health, University of Oxford, Oxford, United Kingdom; Kaohsuing Medical University Hospital, TAIWAN

## Abstract

**Background:**

Delays in patient flow and a shortage of hospital beds are commonplace in hospitals during periods of increased infection incidence, such as seasonal influenza and the COVID-19 pandemic. The objective of this study was to develop and evaluate the efficacy of machine learning methods at identifying and ranking the real-time readiness of individual patients for discharge, with the goal of improving patient flow within hospitals during periods of crisis.

**Methods and performance:**

Electronic Health Record data from Oxford University Hospitals was used to train independent models to classify and rank patients’ real-time readiness for discharge within 24 hours, for patient subsets according to the nature of their admission (planned or emergency) and the number of days elapsed since their admission. A strategy for the use of the models’ inference is proposed, by which the model makes predictions for all patients in hospital and ranks them in order of likelihood of discharge within the following 24 hours. The 20% of patients with the highest ranking are considered as candidates for discharge and would therefore expect to have a further screening by a clinician to confirm whether they are ready for discharge or not. Performance was evaluated in terms of positive predictive value (PPV), i.e., the proportion of these patients who would have been correctly deemed as ‘ready for discharge’ after having the second screening by a clinician. Performance was high for patients on their first day of admission (PPV = 0.96/0.94 for planned/emergency patients respectively) but dropped for patients further into a longer admission (PPV = 0.66/0.71 for planned/emergency patients still in hospital after 7 days).

**Conclusion:**

We demonstrate the efficacy of machine learning methods at making operationally focused, next-day discharge readiness predictions for all individual patients in hospital at any given moment and propose a strategy for their use within a decision-support tool during crisis periods.

## Introduction

‘Patient flow’ describes the flow or movement of patients through the different stages of required hospital care and considers whether they are subject to unnecessary delay [1]. Poor patient flow is especially apparent when incoming emergency department (ED) patients cannot immediately be admitted into the main hospital due to the lack of beds available [2]. However, hospital bed management [3] is frequently reactive and so delays in discharge, and by extension the release of hospital beds, are commonplace [4]. The effects of poor patient flow are amplified during periods of viral infection outbreaks, such as seasonal influenza [5], and the Coronavirus disease 2019 (COVID-19) pandemic [6]. Delays in the release of hospital beds from all patient types lead to hospitals being unable to accept surges of patients arriving with infection. Anticipating the recovery of patients from infections, as well as other illnesses, is therefore a key step in facilitating safer and more efficient releasing of hospital beds, thereby improving overall patient flow in hospitals at times of critically high occupancy.

The recent proliferation of electronic health record (EHR) systems by hospitals provides an opportunity to employ promising data-driven approaches, such as deep learning, to challenging medical problems such as patient discharge prediction [7]. Research in this field to-date has typically focused on classifying, at a single point in time, a patient’s length of stay (LOS) into short, medium, or long stays, a task that is usually performed on admission or pre-operatively [8–20]. Most studies to date have restricted themselves to making predictions for patients of a specific diagnostic category [9–11, 13–16, 19, 21–23]. By contrast, only a small number of studies make more operationally-focused discharge predictions [24–28], out of which four use machine learning (ML) methods [24, 25, 27, 28] and two use deep learning methods [27, 28]. However, these papers restrict themselves in their predictions, to LOS within the intensive care unit (ICU) [27], or to patients who have had a surgical procedure [28], or those that are in certain wards [24].

Our main contributions are as follows:

- Proposal of a strategy for using machine learning models to make operationally focused, real-time discharge predictions for almost all individual patients in hospital at any given time, to improve patient flow in hospital during periods associated with spikes in hospital admissions due to, for example, infection outbreaks such as the seasonal influenza.
- The use of separate models for patients discharge prediction, where independent models are trained independently based on patient admission type and number of elapsed days since admission.
- Feature analysis of variables used within the models; variables learned as being of predictive value can be incorporated in future related studies.

## Methods

### Data

We analysed patient data collected in the EHR of the John Radcliffe Hospital, within the Oxford University Hospitals NHS Foundation Trust, between January 2013 and April 2017, a period that was studied due to the annual resurgence of influenza. This is a teaching hospital group serving a population of 600,000 and providing tertiary services to the surrounding region. De-identified patient data was obtained from the Infections in Oxfordshire Research Database (IORD). One of the largest datasets of its kind, the extracted data contains 431,458 records of unique admissions to hospital from 225,009 de-identified, adult patients.

This study considers a subset of 49,832 admissions, recorded across the four years of the study period, who met the criteria of normal discharge and had full vital-signs observation sets. To select the cohort of patients for which a discharge prediction would be most clinically useful, we considered only patients who are likely to have required a hospital bed. We identified these patients by selecting only patients admitted to general hospital for longer than 6 hours. These 6 hours do not include any time spent in the ED and therefore we do not consider patients who only visited ED. In the UK healthcare system, patients remain under the care of ED for up to 4 hours and only those requiring longer hospital observation or treatment are admitted to main hospital. We also excluded patients attending only as outpatients, for example those attending regular haemodialysis sessions.

Patient admissions were categorised as either planned or emergency admissions, where planned admissions were those scheduled in advance whilst emergency admissions describe patients whose entry into the main hospital was through the ED. While planned admissions are often for surgery, followed by a relatively predictable trajectory of recovery, emergency admissions, which are frequently precipitated by infection, generally present a more challenging patient type for hospital bed managers to predict discharge. The cohort of emergency patients with infection broadly reflects the patient admission type which would spike during a seasonal influenza outbreak, with this cohort having the longest average LOS and with the highest variability in their LOS.

Within our dataset the median (IQR) length of stay was 2.9 (0.85–6.3) days, with Table 1 detailing the LOS variability for the patient cohorts considered. The top ten most presented primary diagnostic codes in the international classification of disease (ICD-10) format, were: J181, I251, N390, I639, S7200, I214, I500, S0650, N179, A419 (lobar pneumonia, atherosclerotic heart disease, urinary tract infection, cerebral infarction, femur fracture, myocardial infarction, heart failure, subdural haemorrhage, acute kidney failure, sepsis). Our predictions were therefore made in a cohort typical of those admitted to hospital, who frequently have complex multifactorial care needs and whose recovery trajectories can be difficult to forecast.

**Table 1:** Statistical analysis of data set.

Patient admission cohort LOS statistics	Planned admissions	Emergency admissions (all)	Emergency admissions (with infection)
Total no. admissions in dataset	11,574	38,258	4,438
Mean LOS (days)	2.3	4.7	5.6
Median LOS (days)	1.9	3.2	4.9
Min LOS (days)	0.25	0.25	0.25
Max LOS (days)	132	195	195
Standard deviation in LOS (days)	5.5	8.3	9.3
IQR in LOS (days)	5.2	6.7	8.4

Total number of admissions and the corresponding statistical summary of LOS for each patient admission.

### Ethics

De-identified patient data was obtained from the Infections in Oxfordshire Research Database (IORD) which has generic Research Ethics Committee, Health Research Authority and Confidentiality Advisory Group approvals (19/SC/0403,19/CAG/0144) as a de-identified electronic research database.

We describe an approach for utilising data from the electronic health records of patients admitted to hospital, to develop models to predict readiness for discharge for patient cohorts within hospital, including those with infection.

### Study design

The system proposed in this work aims to provide operationally focused clinical decision support for periods of crises in hospital. We propose a strategy in which hospital bed managers run these models from within a decision-support tool during a period of high influx of patients with infectious disease. The models would identify the patients who are most likely to be ready for discharge within the next 24 hours. A medical professional would then be assigned to screen the highest ranked patients to confirm the models’ predictions. Once confirmed, hospital bed managers would be able to proactively make discharge arrangements for that patient, to release them from the hospital as quickly as possible and to save valuable time during a critical situation in hospital. Predictions can be made for all patients currently in hospital at any time and thus can incorporate new data as it becomes available. In this study, we simulated predictions being made every 24 hours, with the initial prediction being made on the day of a patient’s admission to main hospital.

**Fig 1:**
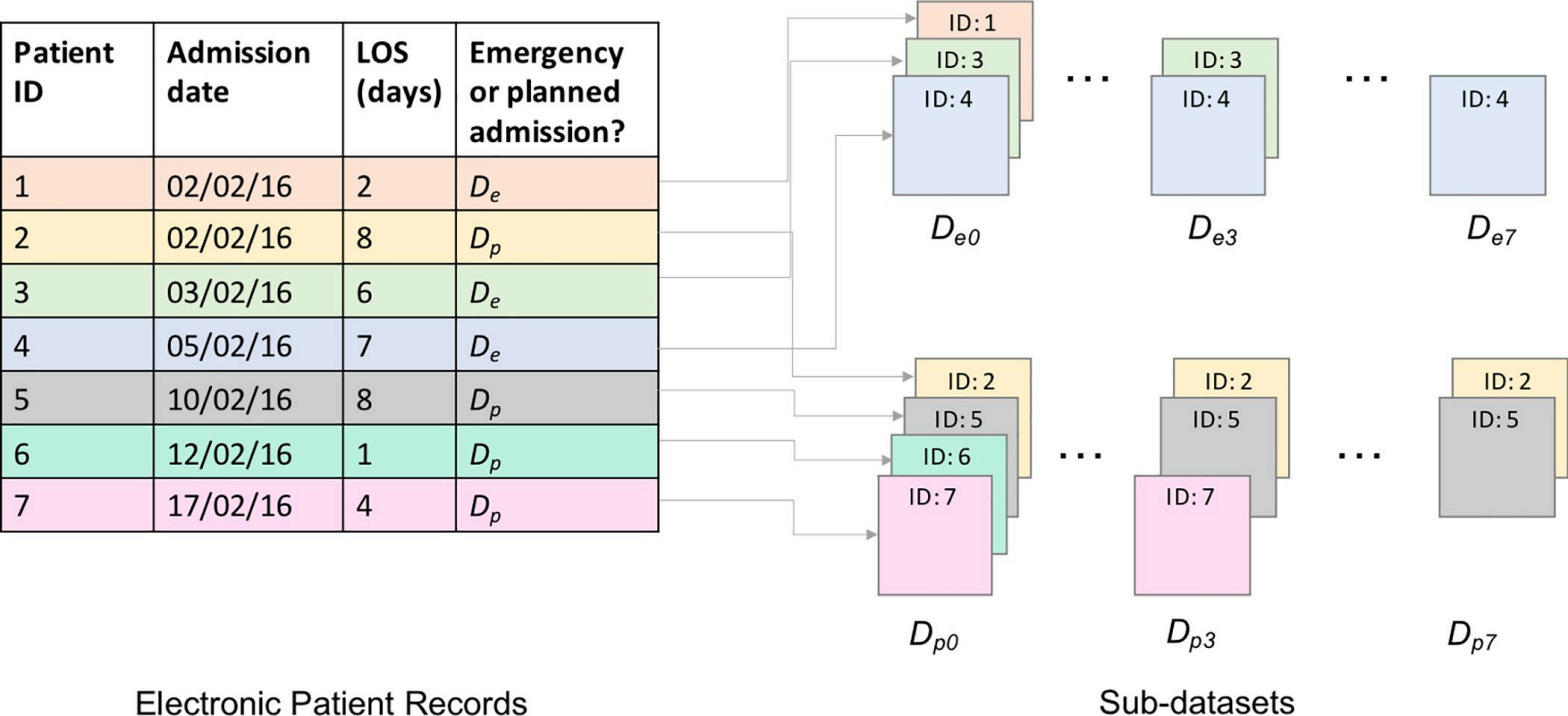
Patient sub-dataset diagram. An illustration of how the sub-datasets were stratified. The figure contains three patients with emergency admissions who had stays that lasted at least 1 day (IDs = 1, 3, 4); at day *t* = 3 only two of the example patients remained (IDs = 3, 4); and on day *t* = 7, only one of these patients remained in hospital (ID = 4), therefore we would only be able to make an 8^th^ day discharge prediction for this remaining patient. A comparable example is also displayed for planned admissions.

We constructed individual models for each patient admission group (planned and emergency admissions) and for each day elapsed since a patient’s admission to hospital. Elapsed times since admission t ∈ {0,1,…,7} were considered, with t = 0 representing the day a patient was admitted to the general hospital. For this study, patient stays were truncated at 7 days. Consequently, 16 different independent models, per model architecture, were developed. The sub-datasets used to train and evaluate the models are denoted *D*_pt_ and *D*_et_, respectively, with the first subscript indicating the patient admission type, and the second indicating the time elapsed in days since admission (Fig 1). For example, as shown in Fig 1, if Patient 1 is a planned patient, who arrives in hospital on 02/02/2016 and stays in hospital for 2 days, they will be included in datasets *D*_p0_ and *D*_p1_. If Patient 3, a different planned admission, arrives in hospital on 03/02/2016 and stays in hospital 6 days, they will also be included in datasets *D*_p0_ and *D*_p1_ along with Patient 1, and will additionally be included in datasets *D*_p2_, *D*_p3_, *D*_p4_ and *D*_p5_.

Each of the sub-datasets were balanced by down-sampling to improve the training and to allow for unbiased testing of the models, details of the down-sampling strategy can be found in Appendix A in (S1 File). The resulting size of each sub-dataset is summarised in Table 2. Diminishing quantities of data were available for increasing *t*, as the sub-datasets only include patients who have not been discharged after *t* days.

**Table 2:** Patient sub-dataset sizes.

*t*	0	1	2	3	4	5	6	7
***D***_**pt**_	11206	5524	1184	972	718	572	478	424
***D***_**et**_	21636	14010	8464	5400	3998	2970	2590	2038

Total amount of unique patient admissions to hospital within each subset of data *D*_pt_ or *D*_et_, in which *t* denotes the time passed in days since the patient’s admission.

In this work, a prediction by a model that a patient will be discharged within the next 24 hours is denoted a *positive* prediction, whilst a prediction that a patient will not be discharged in the next 24 hours is denoted a *negative* prediction. Based on the probability score predicted for each patient, each proposed model ranks patients based on their likelihood of discharge.

### Model development

#### Model architecture

In this study, four supervised ML classifiers were considered. Random forest (RF) and support vector machine (SVM) models, which have previously been shown to give good performance [12, 13, 15, 23, 24] were compared with deep neural networks (DNN) in the form of multilayer perceptron (MLP) models. Logistic regressor (LR) models were also included to serve as a baseline, being a strong comparator from medical statistics. The different classifiers were assessed on their ability to predict whether an inpatient would be discharged within the next 24 hours and the probability scores given by the classifier were used to rank patients in order of their likelihood of discharge.

Model hyperparameters were selected through a nested K-fold cross-validation scheme on the *D*_e0_ dataset, where the outer- and inner-loops consisted of 5 and 3 folds respectively. The 5-fold scheme partitioned the data into training and evaluation folds, whilst the additional 3-fold partition was applied in an inner-loop on the training set folds, to create a training-validation set to assess performance of different hyperparameter choices. A grid-search approach was used to test different hyperparameter combinations, with the combination giving highest average AUROC across all validation folds eventually selected for all models. The hyperparameter values determined and used are detailed in Table 3.

**Table 3:** Model hyperparameters. Table detailing the LR, RF, SVM and DNN models’ hyperparameters.

Model Type	Hyper-parameter	Selection
LR	Norm penalization	l1
LR	Inverse regularisation strength, C	10^3^
RF	Number of trees	75
RF	Minimum samples for node split	2
RF	Minimum samples for leaf node	1
SVM	Kernel function	Non-linear—radial basis function (RBF)
SVM	Soft-margin regularizer, C	10^5^
SVM	Inverse of the variance of the RBF kernel, γ	10^−4^
DNN	Hidden layers activation function	ReLu
DNN	Final layer activation function	Sigmoid
DNN	Hidden layers	2
DNN	Nodes per hidden layer	100
DNN	Dropout rates	0.3
DNN	Weight-initialization	Random, normally distributed weights.
DNN	Optimization algorithm	RMSprop, learning rate of 10^−3^
DNN	Epoch number	150
DNN	Batch-size	20
DNN	Batch normalization	Implemented
DNN	Weight matrix norm constraint	4

#### Feature engineering

Domain knowledge and prior literature were used to determine which information within the dataset would be most useful for predicting patient discharge. Handcrafted features used to train the models included: age, day of the week, procedures information, ICU information and statistical representations of the National Early Warning Score (NEWS) metric [29], which encodes vital signs information, binned into 24-hour periods. Temporal features such as ‘time elapsed since procedure’, ‘time elapsed since ICU discharge’ and features relating to NEWS were populated in ‘real-time’, only being included into the models for which the information would be available. A maximum of 79 features were engineered, the full list of which is summarised in Table 4.

**Table 4:** Table of features.

Category	Features		Total #
Demographic	I. Age	II. Charlson Comorbidity Index (CCI)	2
Seasonal	III. Monday	IV. Tuesday	7
	V. Wednesday	VI. Thursday	
	VII. Friday	VIII. Saturday	
	IX. Sunday		
ICU	X. ICU Ward: CTTC	XI. ICU Ward: AICU	14
	XII. ICU Ward: CICU	XIII. Non-surgical ICU admission	
	XIV. Surgical ICU admission	XV. Planned ICU admission	
	XVI. Unplanned ICU admission	XVII. Reparation ICU admission	
	XVIII. Local ICU admission	IXX. Time elapse since ICU admission	
	XX. Time elapsed since ICU discharge	XXI. Number of ICU admissions	
	XXII. ICU LOS	XXIII. Patient is currently in ICU	
Procedures	XXIV. Time elapsed since last theatre visit	XXV. Number of theatre visits	8
		XXVII. Time elapsed since last procedure	
	XXVI. Patient has been to theatre	XXIX. Patient had a procedure	
	XXVIII. No. of procedures since admission		
	XXX. Patient had radiology-based procedure	XXXI. Time since last radiology procedure	
Bloods	XXXII. Any blood tests since admission	XXXIII. Time elapsed since last blood test	20
	XXXIV. Albumin BT taken in <48 hrs	XXXV. BT taken in <48 hrs	
		XXXVII. Creatinine BT taken in<48 hrs	
	XXXVI. Any BT in <48 hrs abnormal	XXXIX. Potassium BT taken in <48 hrs	
		XLI. White blood cell count BT taken in <48 hrs	
		XLIII. Albumin BT taken in <24 hrs	
	XXXVIII. Sodium BT taken in <48 hrs		
	XL. Urea BT taken in <48 hrs	XLV. Any BT in <24 hrs abnormal	
	XLII. Haemoglobin BT taken in <48 hrs	XLVII. Sodium BT taken in <24 hrs	
		XLIX. Urea BT taken in <24 hrs	
	XLVI. Creatinine BT taken in <24 hrs	LI. Haemoglobin BT taken in <24 hrs	
	XLIV. Any BT taken in <24 hrs		
	XLVIII. Potassium BT taken in <24 hrs		
	L. White blood cell count BT taken in <24 hrs		
NEWS	LII. Mean NEWS since admission	LIII. Max NEWS since admission	26
	LIV. Min NEWS since admission	LV. Variability in NEWS since admission	
	LVI. Most recent NEWS	LVII. First NEWS on admission	
	LVIII. 72–96 hr mean NEWS	LIX. 72–96 hr max NEWS	
	LX. 72–96 hr min NEWS	LXI. 72–96 hr variability in NEWS	
	LXII. 72–96 hr # of observation sets	LXIII. 48–72 hr mean NEWS	
	LXIV. 48–72 hr max NEWS	LXV. 48–72 hr min NEWS	
	LXVI. 48–72 hr variability NEWS	LXVII. 48–72 hr # of observation sets	
	LXVIII. 24–48 hr mean NEWS	LXIX. 24–48 hr max NEWS	
	LXX. 24–48 hr min NEWS	LXXI. 24–48 hr variability in NEWS	
	LXXII. 24–48 hr # of observation sets	LXXIII. 0–24 hr mean NEWS	
	LXXIV. 0–24 hr max NEWS	LXXV. 0–24 hr min NEWS	
	LXXVI. 0–24 hr variability in NEWS	LXXVII. 0–24 hr # of observation sets	
Diagnosis	LXXVIII. Mean LOS of patients in the same CCS category	LXXIX. Variance in LOS of people in the same CCS category	2

A table of all features engineered from the data within the EHR. All features were included in the LR, RF and DNN models. Feature selection was carried out to determine the features to be included in the SVM models from this set. Additional detail about each feature’s data type can be found in Appendix B in (S1 File).

For operational purposes in hospital, it is preferable for a decision support tool to be able to make predictions for all patient groups in the hospital at any given time. Patient diagnosis is typically classified using international classification of disease (ICD) or “Clinical Classifications Software” (CCS) groupings [30], both of which contain too many diagnostic groups to be easily included as ML features directly. As stated earlier, most prior studies restrict themselves to a handful of patient diagnostic categories or a specific patient type. In this study, to directly capture the effects of a patient’s diagnostic category on LOS, features containing the historic mean and variance of the LOS of patients within the same diagnostic category as the patient-under-test were developed. The historic mean and variance of LOS for a particular CCS category were calculated using the training dataset. These mean and variance values were then assigned to patients of the same CCS category in both the training and the test datasets. For patients in the test set with an unseen CCS category, the average of all diagnostic categories was assigned for each feature. Under the present hospital processes, diagnostic categories are assigned and recorded on a patient’s discharge. As such, the information used in this study can be thought of as a proxy for the working diagnosis assigned by clinicians during a patient’s stay. If implemented as a decision support tool, suspected CCS category could be recorded by clinicians and used within the models in real-time.

#### Feature selection

**Fig 2:**
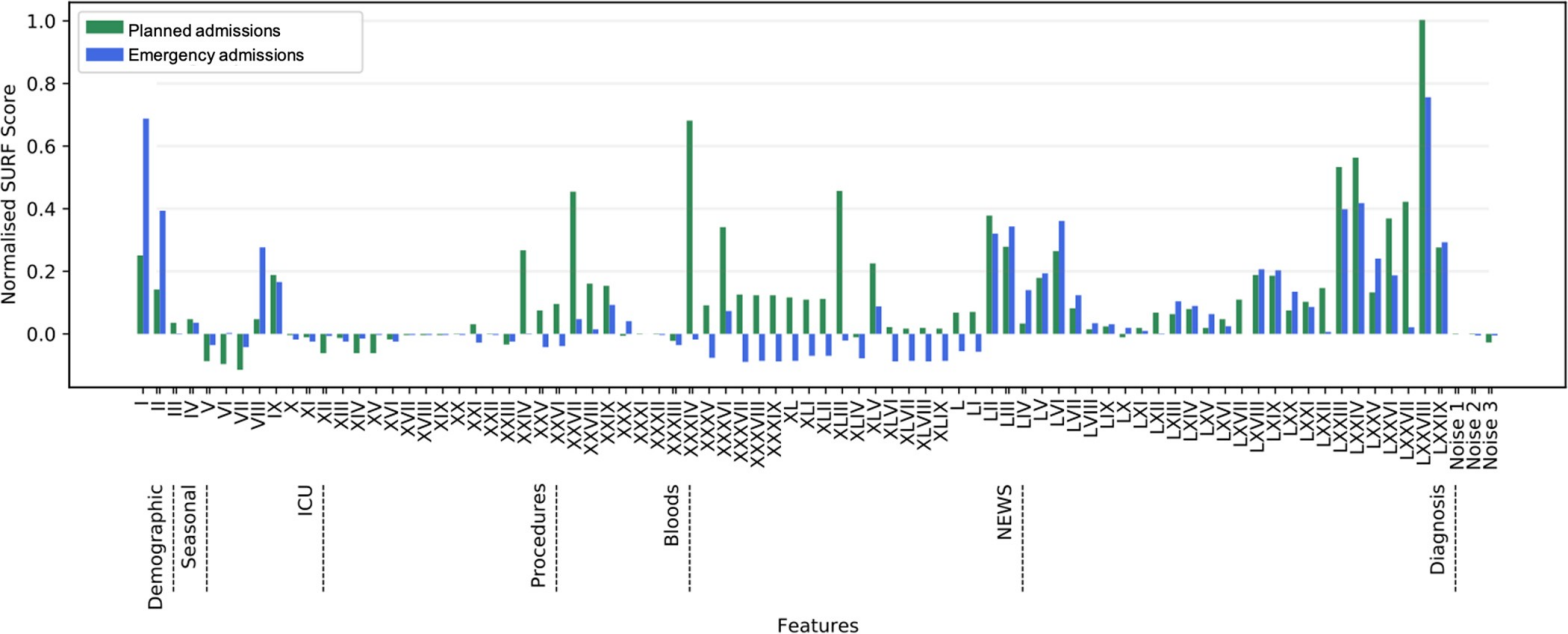
Normalised SURF feature selection scores. The normalised scores resulting from our feature selection methodology, using the SURF feature selection algorithm, for each feature in the datasets. The features on the x-axis of this plot are summarised in Table 4.

For the SVM models, which are particularly sensitive to the inclusion of features with low predictive value, feature selection techniques were applied. Spatially Uniform RelieF (SURF) [31] feature selection algorithm was used to select features, as we found it to be the most robust against white noise features and to be one of the most consistent at picking similar sets of features across 3-fold cross-validation in a comparison between feature selection algorithms. This algorithm uses the proximity of samples in feature space to describe how feature interactions relate to the sample’s class. The normalised scores from running the SURF feature selection algorithm over the engineered features were generated (Fig 2). The detailed methodology of running this algorithm can be found in Appendix C in (S1 File). For the other non-SVM ML models, all features as described in Table 4 were used.

## Results

### Feature importance

Feature selection can provide medical practitioners with valuable insight into the importance of each feature in the predictions made. The results of the feature selection method (Fig 2) show that for both planned and emergency admission types, the feature deemed most important by the SURF algorithm was feature no. 78, the historic mean LOS of patients in the same diagnostic category. This feature, described earlier, aims to capture the effect of a patient’s diagnosis. Age (feature no. 1), Charlson Comorbidity Index CCI (no. 2) and NEWS features (nos. 52–77) were shown to influence discharge predictions significantly, with age and CCI being of particular importance for emergency admissions. For both planned and emergency admissions, abnormal blood test results (nos. 36, 45) were informative. Whether blood tests were taken within the last 48 hour period (nos. 34–42) were seen to be informative features for planned admissions; with albumin blood tests (no. 34) found to be particularly important. This was the only blood test included as a feature which would not be carried out in the hospital by default, but rather would have been requested as an additional test for a patient by a clinician. Information about procedures and operating theatres were shown to be of high predictive value for patients with planned admissions (nos. 24–31), while ICU features (nos. 10–23) were shown not to be of importance for patients in either dataset.

### Predictive performance

In this study, the models developed were evaluated to indicate the efficacy of the models’ use in an operational hospital setting during crises. We propose that, in this setting, 20% of all patients in hospital at a given moment with a positive prediction by the model would be a reasonable proportion of patients to be considered as candidates for discharge. However, this threshold could be adjusted to match the needs of the hospital at any point. Hospital bed managers would oversee the use of these models. We would expect these patients to then have a further screening by a clinician to confirm whether they are ready for discharge or not.

The models were evaluated in terms of their mean and variance in positive predictive value (PPV) over a 5-fold cross-validation. A positive classification was given to any sample with a probability score of 0.5. Each dataset was randomly split into five-folds, with 80% of the data used to train the model, and the remaining unseen 20% used to evaluate the model’s performance on each iteration. PPV represents the proportion of these patients who would have been correctly deemed as ‘ready for discharge’ after having the second screening by a clinician. When computed for the top *x*% of ranked predictions, this metric can be regarded as an evaluation metric particularly well suited to assessing the efficacy of a decision-support tool in clinical practice [32]. For example, if a model achieves a PPV of 0.8, this is equivalent to saying that, for every 10 patients that are prioritised to have a secondary screening by a clinician, 8 patients can subsequently have discharge arrangements proactively made for them, for their release within 24 hours.

The performance of each model, developed for each of the datasets *D*_pt_ and *D*_et_, *t* ∈ {0,…,7}, was evaluated. Moreover, additional analysis on the results of the models trained using emergency admissions *D*_et_ was carried out on the subcategory of these admissions where patients had been diagnosed with infection. This subcategory corresponds to 37 CCS categories. The results for this subcategory are hereafter denoted by D*_et_.

**Fig 3:**
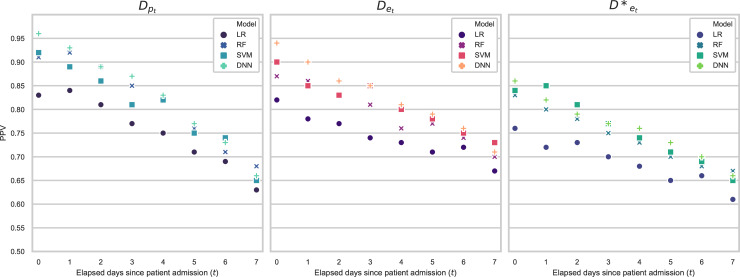
PPV results. Scatter plots giving the PPV, calculated for the 20% of patients with the highest positive classification scores, for LR, RF, SVM and DNN models, trained and tested on the different sub-datasets which reflect the patient cohorts at different points in their hospital stays. Planned admissions are represented by *D*_*pt*_, emergency admissions *D*_*et*_ and emergency admissions with an infectious disease D*_et_.

The mean PPV performance of the different models, calculated for the 20% of patients with the highest positive classification scores within each patient category considered (*D*_pt_, *D*_et_ and D*_et_) are presented across three separate subplots (Fig 3). The mean and standard deviation PPV results, as well as the corresponding NPVs, are presented in Tables 5 and 6.

**Table 5:** PPV results.

			*t* (days)							
Dataset—Models			*0*	*1*	*2*	*3*	*4*	*5*	*6*	*7*
*D*_pt_	**LR**	Mean SD	0.83 0.0077	0.84 0.0091	0.81 0.012	0.77 0.018	0.75 0.025	0.71 0.021	0.69 0.055	0.63 0.079
	**RF**	Mean SD	0.91 0.012	0.92 0.014	**0.89** 0.019	0.85 0.032	0.82 0.046	0.76 0.032	0.71 0.06	0.68 0.094
	**SVM**	Mean SD	0.92 0.0088	0.89 0.0098	0.86 0.0084	0.81 0.017	0.82 0.024	0.75 0.023	**0.74** 0.091	0.65 0.14
	**DNN**	Mean SD	**0.96** 0.0098	**0.93** 0.014	**0.89** 0.013	**0.87** 0.015	**0.83** 0.021	**0.77** 0.024	0.73 0.049	**0.66** 0.10
*D*_et_	**LR**	Mean SD	0.82 0.095	0.78 0.016	0.77 0.013	0.74 0.024	0.73 0.034	0.71 0.031	0.72 0.047	0.67 0.045
	**RF**	Mean SD	0.87 0.012	0.86 0.014	0.83 0.018	0.81 0.023	0.76 0.019	0.77 0.028	0.74 0.034	0.70 0.038
	**SVM**	Mean SD	0.90 0.0059	0.85 0.0094	0.83 0.012	**0.85** 0.015	0.80 0.019	0.78 0.022	0.75 0.016	**0.73** 0.025
	**DNN**	Mean SD	**0.94** 0.0081	**0.90** 0.0083	**0.86** 0.0098	**0.85** 0.0092	**0.81** 0.011	**0.79** 0.018	**0.76** 0.023	0.71 0.029
D*_et_	**LR**	Mean SD	0.76 0.018	0.72 0.022	0.73 0.036	0.70 0.051	0.68 0.047	0.65 0.045	0.66 0.061	0.61 0.067
	**RF**	Mean SD	0.83 0.013	0.80 0.010	0.78 0.029	0.75 0.036	0.73 0.051	0.70 0.057	0.68 0.052	**0.67** 0.067
	**SVM**	Mean SD	0.84 0.014	**0.85** 0.019	**0.81** 0.024	**0.77** 0.023	0.74 0.029	0.71 0.031	0.69 0.034	0.65 0.032
	**DNN**	Mean SD	**0.86** 0.0098	0.82 0.012	0.79 0.021	**0.77** 0.025	**0.76** 0.029	**0.73** 0.033	**0.70** 0.032	0.66 0.037

Table detailing the mean PPV and 1-standard deviation for the LR, RF, SVM and DNN models trained and tested on the different sub-datasets containing planned admissions, *D*_*pt*_, emergency admissions, *D*_*et*_ and emergency admissions with an infection, D*_et_.

**Table 6:** NPV results.

			*t* (days)							
Dataset—Models NPV			*0*	*1*	*2*	*3*	*4*	*5*	*6*	*7*
*D*_pt_	**LR**	Mean SD	0.86 0.0093	0.87 0.017	0.84 0.079	0.79 0.048	0.72 0.055	0.73 0.042	0.70 0.065	0.63 0.071
	**RF**	Mean SD	0.94 0.022	0.95 0.027	0.88 0.019	0.83 0.031	0.81 0.052	0.75 0.077	0.72 0.089	**0.73** 0.084
	**SVM**	Mean SD	0.94 0.0093	0.90 0.011	0.87 0.021	0.84 0.017	0.80 0.025	**0.81** 0.039	0.76 0.082	0.69 0.095
	**DNN**	Mean SD	**0.98** 0.012	**0.97** 0.018	**0.92** 0.021	**0.88** 0.019	**0.85** 0.025	0.81 0.023	**0.78** 0.061	0.73 0.094
*D*_et_	**LR**	Mean SD	0.81 0.0021	0.80 0.032	0.78 0.027	0.77 0.032	0.75 0.044	0.72 0.039	0.74 0.041	0.65 0.048
	**RF**	Mean SD	0.90 0.0072	0.85 0.013	0.86 0.011	**0.84** 0.018	0.79 0.023	0.76 0.031	0.75 0.032	0.69 0.035
	**SVM**	Mean SD	0.92 0.013	0.84 .0011	0.86 0.015	0.81 0.012	0.81 0.017	0.77 0.023	0.74 0.028	0.75 0.031
	**DNN**	Mean SD	**0.93** 0.0091	**0.96** 0.014	**0.87** 0.011	0.83 0.017	**0.82** 0.015	**0.77** 0.023	**0.79** 0.018	**0.75** 0.037
D*_et_	**LR**	Mean SD	0.78 0.026	0.73 0.018	0.72 0.033	0.70 0.045	0.69 0.055	0.64 0.057	0.65 0.042	0.62 0.063
	**RF**	Mean SD	0.84 0.021	0.78 0.023	0.81 0.028	0.77 0.032	0.74 0.029	0.70 0.038	0.69 0.045	**0.69** 0.071
	**SVM**	Mean SD	0.86 0.018	0.83 0.013	**0.82** 0.022	0.79 0.028	0.76 0.024	0.70 0.033	0.67 0.039	0.68 0.041
	**DNN**	Mean SD	**0.88** 0.013	**0.84** 0.011	0.78 0.019	**0.80** 0.021	**0.76** 0.032	**0.73** 0.031	**0.72** 0.038	0.65 0.044

Table detailing the mean NPV and 1-standard deviation for the LR, RF, SVM and DNN models trained and tested on the different sub-datasets containing planned admissions, *D*_*pt*_, emergency admissions, *D*_*et*_ and emergency admissions with an infection, D*_et_.

## Discussion

During outbreaks of disease such as seasonal influenza or the global COVID-19 pandemic, healthcare systems across the world have struggled to cope with an increased demand for hospital beds. This has resulted in situations where patients who required beds in hospital were unable to be admitted, forcing clinicians to make difficult decisions regarding which patients should receive care. Previous work has shown that introducing an ML prediction system can have statistically significant impact on improving overall patient flow [23]. We therefore hypothesize that, with improved patient flow, hospitals would have a greater chance of coping with sudden surges in admissions during crisis periods. However, use of ML techniques to make operationally focused discharge prediction for a broad patient base is an under-researched area, particularly through the use of more advanced ML techniques.

This retrospective study attempts to address these issues through the development of models which are able to reliably classify whether patients will be ready for discharge within the following 24 hours and rank them according to their probability of discharge readiness. The expectation is that these rankings would be used by hospital-bed managers to identify patients to prioritise for a secondary screening. Four different model architectures, LR, RF, SVM and DNN, were compared in their abilities to make this classification. Planned and emergency admissions within the dataset were studied separately, with custom models developed for each. The predictions were made for each day of a patient’s admission, from the first day of their arrival up to 7 days into their stay. It was found that the DNN models often outperformed the other models considered.

Furthermore, we observed that models generally performed best in predicting the discharge of planned admissions, *D*_pt_, rather than emergency admissions, *D*_et_, and were better for predicting the discharge for emergency admissions as a whole, compared to the sub-cohort of emergency admissions with infection, D*_et_. This is likely due to the higher variance in LOS which was present in emergency admissions, and even more so in emergency admissions with infection. A higher variance suggests that by the nature of their admission, these patient groups were less predictable and thus more difficult to classify correctly. Although there were differences in PPV between models developed for the different patient admission types, overall, the results were comparable. This indicates that, if implemented in a hospital setting, we could robustly predict 24 hour discharge readiness for all admission types, and could confidently predict discharge for patients recovering from infection using the models trained on general emergency admission data. This has clear implications during a pandemic. It is also worth reiterating that during periods of crises, the discharge of all patients across hospital is important, as prioritizing one planned-admission type patient for discharge would release a hospital bed for an incoming emergency-admission type patient with infection.

It was seen that PPV is higher and more consistent in models trained and evaluated on datasets where *t* is lower, i.e., datasets for patients with shorter LOS, or earlier into the admission of patients with a longer LOS. This trend could be a combination of two factors. Firstly, a lack of training data (see Table 2) for models with higher *t* is likely to impact performance, particularly for DNN models which generally require more training data than traditional ML models. Secondly, it is possible that it is simply harder to predict next day discharge for a patient who has already been in hospital a considerable length of time, who therefore represents a more complex case. It is also a possibility that the model hyperparameters, which were based on analysis of dataset *D*_e0_ could be overfit to this dataset and not generalize as well to datasets with higher *t*. Nevertheless, if implemented in a hospital setting, it is likely that the performance of the DNN models would improve for datasets containing patients with longer stays as more data is collected.

Lastly, it is worth noting that in general, higher mean NPV results were obtained, which could be interpreted as it being easier to predict when a patient was not ready to be discharged. This can also give us confidence that the models were not making suggestions for patients to be discharged too early, which would be unsafe.

## Limitations

Within the dataset used, there was no information indicating when a patient was medically ready for discharge, therefore the timestamp of the true discharge was used as proxy for this status. Patients who need to be relocated to a subsequent care facility at the end of their stay often have their discharge delayed due to factors out of the hospital’s control [33]. Consequently, the time that they left the hospital is more likely to differ from the time that they were medically fit for discharge. Therefore, as stated earlier, we excluded these patients and restricted our study to only patients who were discharged under normal conditions, to their usual place of residence. A further limitation is that, although this research considered all patients from across a hospital, from different departments and wards, this research was limited to a single hospital. However, prior studies have shown electronic tools to be effective in improving patient flow in other hospital centres, suggesting that the research is generalizable [23, 34]. If implemented in a hospital setting, it would be advised that the hospital records when a patient is medically ready for discharge and that the models should be retrained with this information, and either with data from across multiple centres or with data from the specific hospital where it is intended to be deployed. Furthermore, if not implemented carefully, there is a potential risk that the use of these ML models in hospital could harden any bias in the discharge process. A suggested mitigation of this risk is for hospital bed managers to use the tool, rather than the clinicians directly, thus decoupling the discharge process from clinical prognoses and preventing clinicians from altering their behaviour in response to the models’ output. Finally, this study did not include data from any period associated with a pandemic. Due to the substantial changes within the healthcare system due to COVID-19, this period should be studied separately; research in this area is on-going.

## Conclusion

We have proposed an operationally focused ML classifier which is able to make predictions as to whether a patient will be ready to be discharged within the next 24 hours, for all patients in hospital at any given moment. This classifier is intended to be used during periods that result in a large influx of admissions to hospital, such as peaks in seasonal influenza cases. The intention is for the classifier to be implemented within a well-engineered decision-support tool and for it to be used by hospital bed managers to identify and prioritize patients for discharge. This would improve the efficiency of the safe release of hospital beds and therefore overall patient flow in the hospital. Generally high PPVs were achieved for the top 20% of patients ranked by the models, showing promise that ML systems could prove to be a valuable tool for improving patient flow in clinical settings. Furthermore, variables learned as being of predictive value can be incorporated in future studies which aim to predict real-time discharge or LOS, for individual patients.

## Supporting information

S1 File Supplementary materials. Appendices detailing the data down sampling and feature selection processes used.
(DOCX)
