# Supplementary material for: Improving patient flow during infectious disease outbreaks using machine learning for real-time prediction of patient readiness for discharge
Source: PLoS One. 2021 Nov 23;16(11):e0260476. doi: 10.1371/journal.pone.0260476 (PMC8610279; doi:10.1371/journal.pone.0260476)
Supplement: S1 File — Appendices detailing the data down sampling and feature selection processes used. (DOCX) [file pone.0260476.s001.docx]

**APPENDIX**

1. **Dataset Down-sampling**

Each of the 22 sub-datasets described in Table 1 (*D*_pt_ and *D*_et_, where *t* ∈ {0,…,7}), were balanced to have an equal number of positive and negative classes via random down-sampling. In this context, the process of down-sampling refers to samples (individual patient admissions) of the majority class being removed so that the positive and negative ground-truth classes for the samples remaining were equal in number. This was carried out before the random splitting of the sub-datasets into training and test sets, so that these training and test sets were balanced (i.e. containing an approximately equal number of patients who would be discharged within the 24 hours following a prediction being made, and those who would not). Balancing a training dataset prevents ML models from learning any class imbalances in the data and thus helps to avoid the model making predictions based on a known class imbalance, rather than using what is contained within the features for prediction.

**B Additional Feature Information**

| Category | Features | Data type |
| --- | --- | --- |
| Demographic | I, II | Integer |
| Seasonal | III-IX | One hot encoded {0,1} |
| ICU | X-XVIII, XXIII | One hot encoded {0,1} |
|  | IXX, XX, XXII | Integer (hours) |
|  | XXI | Integer |
| Procedures | XXIV, XXVII, XXXI | Integer (hours) |
|  | XXV, XXVIII | Integer |
|  | XXVI, XXIX, XXX | One hot encoded {0,1} |
| Bloods | XXXII, XXXIV - LI | One hot encoded {0,1} |
|  | XXXIII | Integer (hours) |
| NEWS | LII, LV, LVIII, LXI, LXVI, LXVIII, LXXI, LXXIII, LXXVI | Numerical (continuous) |
|  | LIII, LIV, LVI, LVII, LIX, LX, LXII, LXIV, LXV, LXVII, LXIX, LXX, LXXII, LXXIV, LXXV, XXVII | Integer |
| Diagnosis | LXXVIII, LXXIX | Numerical (continuous) |

**C Feature Selection**

Feature selection methodology using the SURF algorithm. The following steps were taken in order to select features for input to the SVM models:

- - **Step 1:** Three features containing white noise were added to each data-set, *D*_pt_ and *D_et_*, where *t* ∈ {0,…,7}.
  - **Step 2:** An average score for each feature was calculated over 3-fold cross-validation from the scores output by the SURF algorithm.
  - **Step 3**: The value of highest scoring white noise feature was subtracted from all feature’s scores. Thus, a feature with a positive score was deemed to be more informative than white noise.
  - **Step 4:** The scores were averaged across the data-sets *t* ∈ {0,…,7} for each patient admission type and normalised. These scores are shown in Figure 2.
  - **Step 5:** Only features with scores which ranked above white noise on average across the data sets (thus deemed to be more informative than white noise), were included in the SVM model.
